# Nanotechnological innovations in paediatric tuberculosis management: current trends and future prospects

**DOI:** 10.3389/fddev.2023.1295815

**Published:** 2024-01-08

**Authors:** Taiwo Oreoluwa Ajayi, Madan Sai Poka, Bwalya Angel Witika

**Affiliations:** Department of Pharmaceutical Science, School of Pharmacy, Sefako Makgatho Health Sciences University, Pretoria, South Africa

**Keywords:** paediatrics, tuberculosis, pharmacokinetics, pharmacodynamics, oral drug delivery systems, nanotechnology

## Abstract

Paediatric Tuberculosis (TB) continues to be a major global cause of morbidity and mortality. Children are more prone to contracting TB, which can spread quickly to extrapulmonary infection sites. Although the pathophysiology of the disease, drug pharmacokinetics, and the therapeutic window in children differ from those of adults, the same drugs used to treat adult TB have long been utilised to treat paediatric TB infections. Since many current formulations such as tablets are unsuitable for children due to difficulty swallowing and risk of choking, adult medications are frequently used by breaking or crushing tablets to obtain a paediatric dose. This can result in inaccurate dosing due to pharmacokinetic differences in children which could subsequently lead to sub-therapeutic or toxic systemic concentrations. In addition, many of the medications used in the treatment of TB and most medicines in general, have a profoundly unpleasant taste to children causing them to reject and spit out medication which contributes to challenges with adherence, ultimately leading to treatment failure. The aforementioned demonstrates a huge need for the development of novel drug delivery formulations that are paediatric-friendly and address the limitations of current dosage forms. This review discusses the currently available oral paediatric formulations, recent developments of novel oral drug delivery systems studied to overcome the current problems associated with the treatment of tuberculosis in paediatrics and provides potential direction for future research through nanotechnology by using a SWOT analysis.

## 1 Introduction

According to estimates, 10.6 million persons contracted TB in 2021, an increase of 4.5% from 10.1 million in 2020 (World Health Organization, 2022a). Young children, aged <2 years, represent a distinct risk category due to their greater risks of contracting TB if infected, with rapid disease progression, and high case fatality rates even though the majority of TB deaths occur in older age groups (Marais et al., 2004). Among the estimated 10.6 million people infected with TB in 2021, an estimated 11% of the total TB cases were children (World Health Organization, 2022b). The majority of paediatric TB deaths occur in low to middle-income nations (Southeast Asia and African regions), mostly in children under the age of five who frequently die before receiving a TB diagnosis (Martinez et al., 2019).

Tuberculosis is a highly contagious, air-borne and chronic illness that is caused by a bacteria called *Mycobacterium tuberculosis* (Mtb). Despite TB being a primarily pulmonary infection, it can also cause damage to the brain, intestines, kidneys, or spine (Zaman, 2010). Clinically, TB is characterised by persistent cough, the production of sputum, inappetence, loss of weight, night sweats, fever and haemoptysis (Loddenkemper et al., 2016).

Despite both adults and children being generally at risk for contracting TB, children face a higher likelihood of infection from household contact with infected adults, most of whom are their guardians. Children are more at risk of contracting the infection due to their underdeveloped immune system which could be even worse in children born with HIV/AIDS. A research study revealed that effective *Bacillus* Calmette-Guerin (BCG) vaccination initiatives not only decrease the prevalence of latent TB infection in children but also lower the occurrence of active TB (Styblo and Meijer, 1976). Poor immunisation status (unvaccinated with BCG) is a risk factor linked to TB infection, other risk factors include, malnutrition, poverty, immunocompromised, lack of parenteral knowledge about TB, overpopulation, exposure to infected adults, consumption of unpasteurised milk, as well as chronic illnesses (Joseph Attah et al., 2018).

Currently, TB therapy is lengthy, takes a uniform approach for different ages, and frequently includes drugs that are not always accessible in paediatric-friendly dosage forms despite the wide variety in TB pathophysiology and presentation in the paediatric population (Sandra et al., 2008). Regarding the pathophysiology of TB in children, the immune system in children is still developing, and they may have a less mature immune response compared to adults. Furthermore, the disseminated forms of TB is more common in children than in adults as adults present with more pulmonary symptoms (Williams, 1919). The identification of the Mtb strain (drug-sensitive or resistant) that causes the disease plays an important role in the selection of a particular medication regimen for paediatric patients. Generally, the dosage is derived from adult doses but due to the fact that children and adults have different biopharmaceutical, physiological and developmental characteristics, paediatric doses must be modified to account for the age, weight, liver and kidney function of the child (Pai et al., 2016). The age diversity of the paediatric population calls for the use of a variety of dosage forms (intravenous, intramuscular, inhalation and rectally), with the oral route as the preferred option for paediatric patients (Alyami et al., 2017; Galande et al., 2020). Liquid dosage forms are suitable for patients under the age of two. Solutions, emulsion, suspension, and effervescent are acceptable for children in the range of 2–6 years old. The most appropriate dosage forms for children between 6 and 12 years old are orodispersible and chewable tablets. However, taste issues may exist when it comes to oral medications (Riet-Nales et al., 2016).

One of the key factors in patient compliance with medications, particularly for paediatric patients, is palatability. A number of techniques, such as the use of sweeteners (Bajaj and Kale, 2016) and excipients that mask the bitterness of drugs (Andrews et al., 2021) and taste enhancers, active pharmaceutical ingredient alterations (Pimparade et al., 2015), solubility (Nguyen et al., 2018), and nanotechnology (Krieser et al., 2020), are suggested for taste enhancement and modification. The several advantages of nanotechnology in the pharmaceutical industry include improved therapeutic effectiveness, targeted drug delivery, and reduced toxicity. Nanocarriers are better formulations for paediatric patients because of these benefits (Farjadian et al., 2019). Although there have been developments of age-appropriate oral medication delivery systems such as sprinkles, suspensions and powders for reconstitution, studies that address nano-formulations for application in paediatric treatment have been poorly studied.

The importance of organic nanomaterials such as liposomes and polymeric nanostructures, including dendrimers and micelles, and their role in the development of targeted drug delivery is well established (Mosa Mubarak and Ahmad, 2020). Several articles have addressed the treatment of TB using nanotechnology-based delivery systems (Banyal et al., 2013; Patricia Bento Da Silva et al., 2016a; Nasiruddin et al., 2017a), however, they only focus on treatments convenient for adult administration and have not addressed the potential use of nanotechnology in the treatment of paediatric TB.

This study will focus on the current strategies for the treatment of TB as well as child-friendly formulations for paediatric use and a review of recent developments and future strategies for nano-based oral paediatric drug delivery strategies. Studies on oral nanostructures for the treatment of TB that have been explored will be discussed. Finally, a SWOT analysis of oral nano-based drug delivery systems will be outlined.

## 2 Aetiology of tuberculosis in paediatrics

**FIGURE 1:**
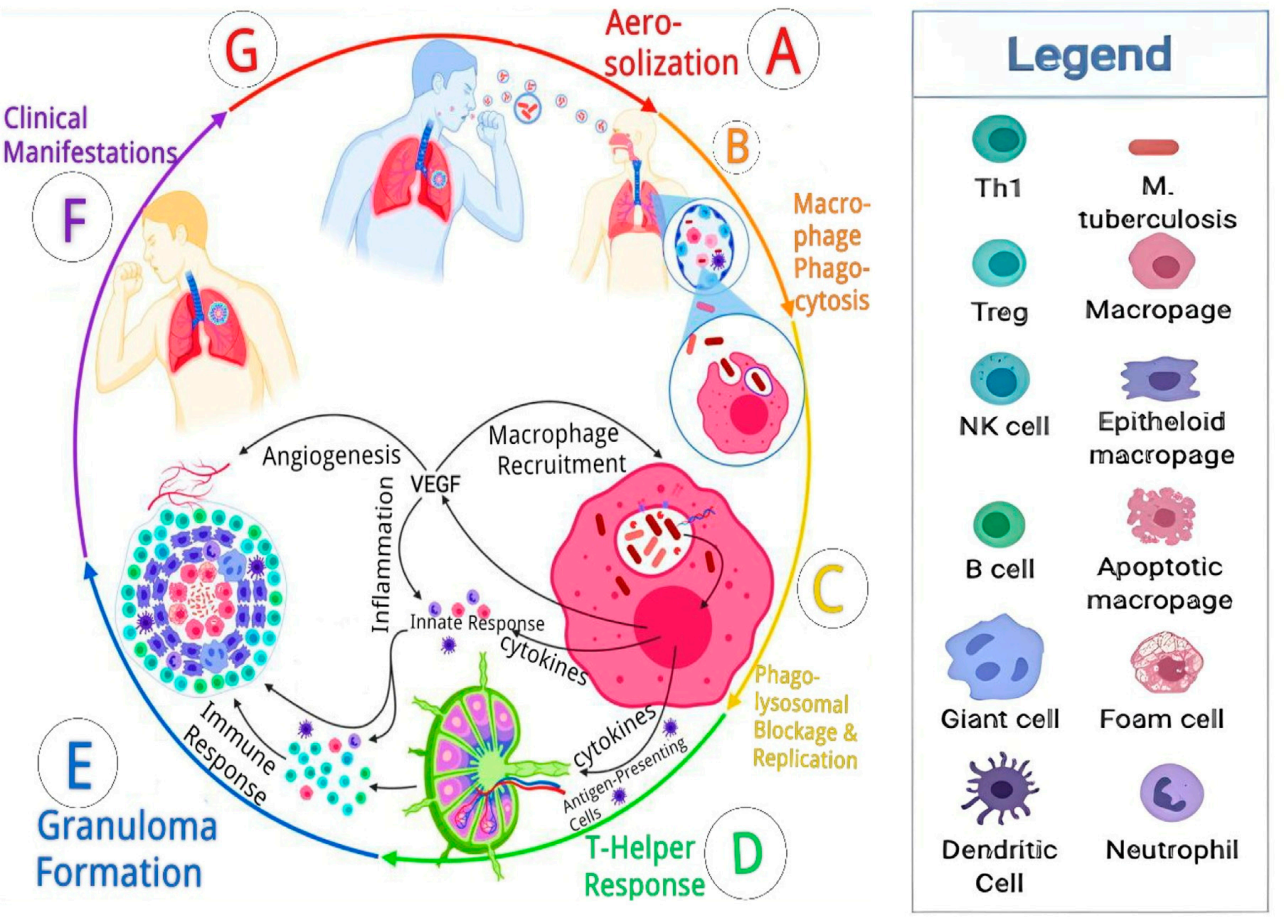
Pathophysiology cascade of tuberculosis. Reproduced from David (2022). With permission from Elsevier Journal of Clinical Tuberculosis and Other Mycobacterial Diseases Copyright 2022.

Mtb is spread via the respiratory route when small (1–5 μm) infected droplets are released from individuals with laryngeal or pulmonary TB and breathed into the alveoli by close contacts (Smith, 2003; Tania, 2017; David, 2022). Pulmonary TB is more prevalent and can lead to transmission of the disease through coughing, while laryngeal TB, affecting the voice box, might cause specific voice-related symptoms and usually does not contribute to disease transmission to the same extent as pulmonary TB. Alveolar macrophages absorb the Mtb after inhalation, and they create caseating granulomas to house the bacilli. Some bacilli are transported by macrophages to the local lymph nodes. The bacilli can move from the local lymph nodes directly into the systemic bloodstream or through the lymphatic duct before a sufficient immune response is generated. If the spreading infection is not stopped by forming an acquired immune response, TB illness spreads widely. The following processes are involved in the spread of the infection: aerosolization, macrophage phagocytosis, phagolysosome replication, formation of granuloma, clinical signs and symptoms, and transmission (Maison, 2022a). Moreover, extrapulmonary TB, which represents 20%–30% of paediatric TB cases, similarly receives its seed organisms from occult dissemination (Young Highsmith et al., 2019). Aerosolization marks both the initiation and culmination of the TB pathophysiological process. This occurs when an individual with active TB forcefully expels air, as seen in actions like coughing. Subsequently, a susceptible person inhales the aerosolized Mtb and small droplets capable of reaching the alveolar sacs, where they encounter macrophages, dendritic cells, and monocytes. Upon inhalation, macrophages phagocytose the bacteria in an attempt to eliminate the invader, while dendritic cells migrate to lymph nodes to activate T-helper cells. However, Mtb thwarts phagolysosome fusion, avoiding destruction, initiating replication, and releasing DNA, RNA, proteases, and lipids. Concurrently, the macrophages release cytokines and vascular endothelial growth factor (VEGF). VEGF triggers angiogenesis, enhancing vascularization to the lesion, while cytokines initiate the innate response, recruiting natural killer (NK) cells, dendritic cells (DC), neutrophils, and macrophages in various forms. The T-helper cell response involves the migration of TH1, Tregs, and B cells, primed in the germinal centre, culminating in the formation of the granuloma. This granuloma acts as a containment structure, isolating the bacteria and preventing systemic spread. However, in cases of immunocompromise, the granuloma fails to contain the bacteria, leading to their proliferation and the emergence of diverse clinical manifestations. In later stages or under conditions of immunocompromise, the compromised granuloma allows the bacteria to spread. During this phase, the infected host, who was originally susceptible, can aerosolize the bacteria, initiating the cycle anew (Azman et al., 2014; Gavin et al., 2017; Maison, 2022b). The pathophysiology of TB is depicted in Figure 1.

Children under the age of two, particularly newborns, are most susceptible to contracting TB infection as a consequence of their underdeveloped immune systems. Due to the fact that they moved from the purportedly sterile environment into the antigen-exposed world, they typically lack protection from damaging inflammatory responses (Hamzaoui et al., 2014). The innate immune response to TB begins with the alveolar macrophage, which is crucial in boosting the body’s defences against infection. There is less microbial death and monocyte activation at the site of infection in infants compared to adults, according to *in-vivo* studies conducted (Brook et al., 2017). Therefore, mycobacteria may be able to negate the effects of the innate immune system in the newborn and infant before the start of an immune response directed against an antigen (Newton et al., 2008).

## 3 Current recommended guidelines for paediatric TB, multi-drug resistant TB, extensive drug resistant TB

The basic goal of anti-TB therapy is to eradicate TB infection from the patient resulting in an avoidance of death or any late effects. Furthermore, there is an intention to prevent the recurrence, spread and development of resistance of the bacteria to currently available treatments while offering the least occurrence of toxicity possible (Nahid and Hopewell, 2008; Rossiter, 2020). Currently, the first-line drugs generally administered for drug-sensitive TB include isoniazid (INH), rifampicin (RIF), pyrazinamide (PZA), ethambutol (EMB) which are given for 2 months and thereafter RIF and INH for 4 months. Second-line drugs are used in the case of RIF resistance, multidrug resistance TB (MDR-TB) and extremely drug resistance (XDR-TB) (Rossiter and University of Cape Town, 1998). They can be classified into four groups as summarized in Table 1.

**TABLE 1:** Current recommended daily drug doses for Paediatric tuberculosis (South African Medical Association, 2020).

Drug	First line -daily dose (mg/kg/day)	
	Intensive phase: 2 months	Continuation phase: 4 months
RIF	10–20	10–20
INH	7–15	7–15
PZA	30–40	
	Second line	
Levofloxacin	Group 1 15–20	
Moxifloxacin	>8 years: 7.5–10	
Gatifloxacin	800 mg/day	
Amikacin	Group 2 15	
Capreomycin	IM, 15–20	
Kanamycin	15	
Streptomycin	IM, 15–20	
Ethionamide	Group 3 15–20	
Cycloserine/terizidone	15–20	
Linezolid	10–15 (>10 years)	
Clofazimine	2–5	
Bedaquiline	Group 4 6 (14 days) 3–4 mg/kg/3x per week (6 months)	
Delamanid	50 mg BD (>6 years, >20 kg)	

Group one includes fluoroquinolones such as gatifloxacin, moxifloxacin (MOX) and levofloxacin. Second-line injectable agents such as kanamycin, capreomycin, streptomycin and amikacin are group two. Other core second-line agents ethionamide, cycloserine, terizidone, clofazimine and linezolid are another group. Group four agents are add-on agents which are not part of the MDR-TB regimen are PZA (30–40 mg/kg/day), EMB (20–25 mg/kg/day), high-dose INH (15–20 mg/kg/day), bedaquiline and delamanid (Kalaiselvan et al., 2021).

Treatment of paediatric patients with drug-sensitive TB involves taking a combination of anti-TB drugs for 6 months or longer, if necessary, especially as resistance occurs. Children with drug-susceptible TB are treated with the same conventional medications as adults (World Health Organization, 2022c). When treating drug-susceptible infection in children an intensive phase consisting of RIF (10–20 mg/kg with a maximum dose of 600 mg/day), PZA (30–40 mg/kg), INH (10–15 mg/day with a maximum dose of 300 mg/day), and EMB (15–25 mg/kg) generally form conventional treatment regimen. Following this, patients receive INH and RIF in the aforementioned doses for 4 months (South African Medical Association, 2023).

The majority of the medications administered in the intensive therapy phase are effective in eradicating the bacilli with the main goal of promoting disease regression, halting the spread of infections, and preventing the emergence of drug resistance. The sterilising agents used in the continuing phase of treatment primarily destroy dormant or infrequently metabolising bacilli, which frequently leads to complete bactericidal effects, disease regression, and relapse prevention (Schaaf et al., 2015). It is typically quite difficult to determine the ideal TB antibiotic dose needed to achieve steady-state concentrations that would improve effective therapy without producing toxic blood concentrations in children and adolescents. This frequently results in failed therapy and subsequently, drug resistance, which becomes more challenging to successfully manage. Treating the disease at this stage becomes complex due to several factors, including the prolonged duration of treatment and heightened costs (Maphalle Lehlogonolo. et al., 2022a).

With the addition of novel reformulated drugs, the implementation of shorter regimens, and a transition to predominantly all-oral MDR-TB treatment regimens, MDR-TB treatment has evolved significantly in recent years (World Health Organization, 2020). The treatment durations should be adjusted depending on the gravity of the disease, the level of drug resistance, and the site of infection. They can range from 9 to 18 months in children with TB, which is frequently non-severe and paucibacillary (Seddon et al., 2021).

## 4 Pharmacokinetic and pharmacodynamic diversity in children

As a result of the wide range of pharmaceutical and clinical factors that have to be taken into account to assure the safety, quality, and effectiveness of the finished product, developing an age-appropriate formulation is a difficult undertaking. Developing paediatric formulations is especially challenging due to the distinct requirements and needs of this target population compared to adults. Dose flexibility is necessary to accommodate the dosing needs of all age brackets because a drug’s pharmacokinetic (PK) and pharmacodynamic (PD) profile differs significantly depending on a child’s developmental stage (Batchelor and Marriott., 2015a). Excipients that are typically thought of as harmless could pose a safety risk to children, which would require further thought throughout the formulation process. Because of their developing physiology, children may react differently to excipients than adults. Age, weight, and organ function variation must all be considered during formulation. While some excipients appear to be harmless in adult populations, they may pose health challenges for paediatric patients due to their unique metabolic processes and vulnerabilities (Salunke et al., 2013).

Paediatric patients with active TB illness continue to experience unfavourable pharmacotherapeutic outcomes due to inaccurate antibiotic administration. Regarding safety and effectiveness, it is anticipated that the prescribed dose of anti-TB medications in paediatrics will produce a pharmacokinetic profile that results in systemic effects comparable to those of adults exposed to equivalent drug levels. However, the maximum concentration (C_max_) and the area under the concentration-time curve (AUC), two measures used to estimate the ideal dose, frequently fail to produce repeatable results in both adults and paediatric populations. Although there is a strong association between C_max_ and AUC in various adults, children typically have AUC values that are lower than those of adults despite having similar C_max_ values to adults due to their quick drug metabolism and clearance. This justifies the necessity to alter paediatric doses in accordance with the child’s body weight and growth phase, as defining a paediatric dose targeting adult C_max_ or AUC results in significantly varied doses and therapeutic outcomes (Mckenna, 2019). To learn more about TB diagnostics among Tanzanian children susceptible to TB, a longitudinal cohort study was carried out. The goal was to measure the estimated peak TB drug concentrations. According to the study’s findings, using TB medications with proper co-formulations and established bioavailability is a crucial step in maximising drug exposure. The results of the study indicated that there is a greater need for thorough paediatric PK/PD studies to identify the best doses and exposure targets in relation to treatment effectiveness and the potential to shorten treatment duration (Justine et al., 2020).

All drugs given intravenously have immediate access to the systemic bloodstream, with the exception of those that are formulated as prodrugs. In contrast, when various drug delivery methods such as enteral, subcutaneous, intramuscular, and inhalational are used, the PK parameters related to absorption (both rate and extent) is significant. The majority of medications given to newborn and paediatric patients are administered orally as it improves ease of administration and offers more flexible dosing for the prescriber. Specific drug physicochemical properties, such as drug hydrophilicity, solubility and permeability, as well as physiological parameters such as gastric acidity, intestinal transit time, drug-metabolising enzymes, and drug transporters as well as external factors (diet, co-morbidity, nutritional status) affect to what extent a drug is absorbed through the gastrointestinal tract. With growth, absorption, distribution, metabolism and elimination may alter significantly, reflecting changes in the paediatric patient’s ability to absorb drugs (Merchant et al., 2016).

Once a drug has entered the systemic circulation, it will begin to spread across various tissues or organs. The distribution pattern will be influenced by both physiological (such as protein binding and tissue uptake) and physical (such as lipophilic or hydrophilic, level of ionisation) processes. Protein binding, pH, systemic and local blood circulation, permeation of innate “barriers” (such as the placenta and blood-brain barrier), and body composition all affect distribution (Batchelor and Marriott, 2015b). The physiological areas into which a drug will disperse change due to maturational changes in body composition with age. When compared to older children and adults, neonates and infants have a larger proportion of body water relative to body weight, and preterm neonates exhibit a more elevated ratio when compared to full-term neonates (Allegaert et al., 2017). When drugs are delivered based on weight, newborns and babies have greater extracellular and total body water compartments, which leads to reduced drug concentrations in the bloodstream that disperse into these respective regions (Bartelink et al., 2006). For lipophilic drugs, the opposite is true. A water-soluble drug’s volume of distribution in newborns and throughout children serves as an example of this trend. Contrarily, when compared to children or adults, a lipophilic molecule, displays a correspondingly reduced distribution volume (Anker et al., 2018). Drug distribution is greatly influenced by protein binding. In particular, preterm neonates are at higher risk as a result of altered interactions of drugs with proteins. As a consequence of reduced levels of total protein and albumin in the plasma, newborns show diminished drug binding to protein. Alpha1-acid glycoprotein binds primarily to basic drugs, whereas albumin primarily binds to acidic medications (Nwobodo Ndubuisi and Obu Herbert, 2015). Even though other systems such as the kidney, gastrointestinal tract, lungs, and skin contain drug-metabolising enzymes, the liver is the primary organ for drug metabolism. The developmental pattern of enzyme expression involved in phase I and phase 2 metabolism can profoundly influence a drug’s PK, therapeutic efficacy, and safety profile in children (Hines, 2008). Children’s lower relative renal tubular absorption and secretion compared to adults’ lower glomerular filtration rates may lead to a slower rate of drug clearance and, thus, a higher chance of toxicity (Lu and Rosenbaum, 2014). Although the underdeveloped glomerular filtration and renal tubular system limit the excretion of numerous drugs that are eliminated through urine in unchanged form during the neonatal period and early infancy, it has been noted that many drugs are eliminated from the plasma at a similar or even higher rate than in adults in late infancy and/or in childhood (Strolin et al., 2005). Pharmacodynamics refers to the physiological and biological reactions triggered by the drug and is generally distinct from pharmacokinetics, whereas pharmacokinetics encompasses processes such as absorption, distribution, metabolism, and elimination of medicines which can be accessed through blood/plasma sampling. For logical dosing, it is essential to understand the connection between children’s pharmacokinetics and pharmacodynamics. Utilising biological samples, pharmacokinetic analysis measures changes in drug concentration over time. Validated endpoint metrics for children are required for pharmacodynamic analysis (Batchelor and Marriott, 2015c).

Apart from their body mass, nutritional status and maturing enzyme functions, children’s pharmacokinetic variability is also influenced by HIV infection. For instance, when adults received a double dose of lopinavir/ritonavir, it resulted in sufficient levels of lopinavir but in children younger than 3 years, suboptimal concentrations were observed, indicating that the interaction between RIF and lopinavir/ritonavir differs in young children and adults (McIlleron et al., 2011). HIV infection can affect the function of organs involved in drug metabolism. HIV infection has been linked to impaired absorption of anti-TB drugs like rifampicin within the gastrointestinal tract (Charles et al., 1996; Burman et al., 2001; Gurumurthy et al., 2004). This alteration in organ function can lead to changes in the clearance and elimination of medications from the body (Nicola et al., 2020). RIF and raltegravir’s interaction is another illustration. While adults showed effective exposure when administered standard dosed raltegravir (Anne et al., 2015), it required doubling the dose in children to counter the interaction with rifampicin (Meyers et al., 2019). These examples illustrate that drug-drug interactions and pharmacokinetics should be studied in children from various paediatric groups before applying adult regimens for treatment.

## 5 Oral paediatric formulations for TB treatment

In order to improve the formulations of currently available antitubercular drugs, make them easier for paediatric patients to use on a daily basis, enhance drug levels at target sites, improve therapeutic index, minimise harmful effects, and shorten the duration of treatment, scientists are constantly looking for new drug delivery strategies (Maphalle et al., 2022b). Due to their ongoing development, paediatric patients need a variety of oral drug delivery systems compared to other demographic groups, which has implications for dose and administration. Oral drug administration, such as liquids or dispersible tablets is convenient, economical, and safe for treatment in children (Rampedi et al., 2022a). Since conventional formulations were not created for this patient population, compounding and manipulation have become standard procedures. Therefore, there is a need for the development of oral drug delivery techniques that are specifically designed for the unique requirements of the paediatric group. Elderly patients could potentially gain advantages from formulations that prioritize patient-specific approaches aimed at addressing challenges related to compromised physiological functions, visual impairment, motor skills, and swallowing abilities, ultimately improving adherence and consistency (Lopez et al., 2015). The oral method continues to be the most favoured, particularly for long-term therapies. There is no discomfort and no need for expert staff. However, because the majority of oral formulations are solid, it might be difficult for young children to swallow them and modify their dosage. In the treatment of newborns and babies, where liquid formulations are generally advised, this factor is significant (Sosnik et al., 2012).

## 6 Liquid formulations

Liquid formulations for oral use often seen in pharmaceutical practice fall into two categories: monophasic or bi-/polyphasic. Monophasic systems comprise a single homogeneous phase, such as solutions, mixtures, elixirs, tinctures and syrups. In contrast, bi-/polyphasic liquid dosage forms encompass two or more distinct phases, like emulsions and suspensions (Hood, 2020). Part of the numerous formulations that enable the administration of medications orally include liquid formulations (Frutos et al., 2020). Liquids provide maximum dosing flexibility, and a single formulation can be used across a variety of age groups (including neonates). However, the patient must be satisfied with the volume utilised, and the dosing apparatus must be suitable for the intended application (Batchelor and Marriott, 2015d). Although liquid TB formulations such as rifampicin suspension (Rifadin^®^) are primarily used to treat paediatric patients, they can also be utilised to treat patients who are prone to overmedication in liquid form. For paediatric patients, they are highly helpful since they are more suited to the patient’s characteristics, make drug administration easier, and allow more dosage flexibility to accommodate the patient’s weight and age. Oral liquid formulations, however, have a number of drawbacks from a galenic perspective, including shorter shelf life and less stability than solid oral preparations, physicochemical incompatibility issues, microbial contamination and sometimes poor taste (Frutos and Negre, 2020). For the formulation of compounds with bad tastes, suspensions may be particularly helpful since they can make the formulation more palatable by reducing the amount of medication in solution. Additionally, suspensions allow for a larger drug loading than solutions, which lowers the dose volume. To alter medication release, suspensions with coated pellets or ion exchange resins could be helpful. However, solutions are favoured over suspensions due to greater oral acceptability of taste, and suitable drug delivery properties. Additionally, suspensions must have enough information on labelling to guarantee proper dosing (EMEA, 2005). There are very few liquid anti-TB medications available. The majority of first-line medications are only produced extemporaneously and are not commercially available in paediatric dosage forms (Sosnik et al., 2010a).

Two stable extemporaneous liquid formulations of delamanid were developed as part of a study to enable dose titration and administration to patients who have difficulties ingesting tablets. These formulations were made to be simple to prepare in a pharmacy or dispensary, including in low- or middle-income countries, using readily available, affordable ingredients and minimal equipment, and remain stable at ambient conditions up to 30°C for several weeks. The formulations could be made on the spot and kept for 15 days in simple syrup and 30 days in the sugar-free mixture. They had the potential to significantly increase children’s access to delamanid and could be used to treat RIF-resistant TB in patients of all ages who have trouble swallowing pills, including adults and children (Taneja et al., 2023a).

Taneja et al. (2023b), again, achieved the development of a 10 mg/mL clofazimine formulation in syrup and sugar-free carriers using readily available excipients. In the syrup formulation, the potency of clofazimine remained within the range of 90% and 110% for a duration of 30 days, while in the sugar-free formulation, it stayed within the same range for a duration of 15 days. These formulations could make it possible for patients—including children—to receive clofazimine readily and facilitate more precise dose titration.

Stability of INH, PZA, and RIF in liquid form were studied under *in vitro* conditions that mimic the gastrointestinal tract of paediatric patients, the study included both individual assessment and examination within a fixed combination at paediatric doses. The study examined the doses for individual drugs, or the fixed combination designed for paediatric use in order to identify the pH at which maximum stability occurs. The effects of ascorbic acid and hydroxypropyl-cyclodextrin on the formulation’s stability and solubility of rifampicin were investigated. A formulation was created that combined the minimum dissolved dosages for children for both isoniazid and rifampicin as recommended by the WHO (Santoveña-Estévez et al., 2020). Table 2 summarises commercially available formulations for the treatment of TB in paediatrics.

**TABLE 2:** Liquid formulations available for Paediatric tuberculosis.

Drug	Dosage form	Trade name
Levofloxacin	Solution	Levaquin^®^
Rifampicin	Suspension	Rifaldin^®^/Rimactazid Paed^®^(dispersible in water)
Linezolid	Suspension	Zyvox^®^

## 7 Solid preparations for reconstitution

In the development of paediatric formulations, the incorporation of powders, reconstitutable granules and dispersible tablets, is a common technique because solid drugs often have more stability than liquid formulations. These reconstituted medicines, however, also need to have their tastes masked. Depending on the product, reconstitution can take place at the site of administration or before dispensing (Batchelor and Marriott, 2015e). These medications should always be completely dissolved before administration; nevertheless, this may need considerable amounts of diluent, which could be problematic for young patients. Furthermore, they may not be appropriate for all patients, such as those with renal dysfunction, as they typically contain higher sodium and/or potassium ion concentrations (EMEA, 2015). As they offer versatility in medication administration, oral solid dosage forms platforms are able to offer child-friendly preparation choices to all paediatric populations and geriatric patients. To increase patient compliance in children, these formulations can serve as a foundation for long-acting medications and fixed-dose combinations of multiple medications (Ch and Kandasamy, 2018).

In a study by Rampedi et al. conducted a study in which they found that a novel multi-particulate reconstitutable suspension formulation, containing fixed doses of first line anti-TB drugs (150 mg rifampicin/300 mg pyrazinamide per 5 mL) holds promise as a TB treatment option for in the paediatric population. The optimised product was a reddish-brown, easily flowing, finely structured powder that showed optimal reconstitution and settling characteristics when mixed in an aqueous solution. The new drug-loaded suspension formulation had no substantial adverse effect on the model cells (HepG2) tested and was possibly biocompatible (Rampedi et al., 2022b).

The development of orodispersible films for long-term INH chemoprophylaxis for the paediatric population was the goal of a study by Chachlioutaki et al. The orodispersible films were made of nanofibers that had sufficient thermal stability and might amorphize drugs. Nanofibers are engineered to rapidly absorb saliva, dissolve, or break down inside the patient’s mouth without requiring chewing or drinking. This quick breakdown facilitates the immediate release of medications onto the buccal mucosa, enabling rapid absorption (Erick et al., 2018). An *in vitro* test revealed that the orodispersible films instantly disintegrated in under 15 s upon exposure to salivary fluid, leading to a fast and complete release of INH in under 60 s. This characteristic ensures a child-friendly drug administration method (Chachlioutaki et al., 2020).

In another study, a polymer-based, orodispersible film formulation containing PZA was designed, optimised, and subjected to a thorough *in vitro* evaluation as a potential replacement for flexible paediatric dosing. The solvent casting technique was used to formulate the PZA-loaded orodispersible matrices. A combination of pharmaceutical excipients, including a copolymer of polyvinyl alcohol and polyethylene glycol as base material, citric acid as a preservative, and xylitol as a paediatric-friendly sweetener, were used to create the PZA-loaded formulation. According to findings from physicochemical analysis and stability tests, the orodispersible drug formulation demonstrated thermodynamic and environmental stability in specified storage conditions. Preliminary investigations involving taste evaluations and *in-vitro* assessments indicated that the drug formulation was pleasant tasting, easily administrable, and biocompatible within tested conditions. The oro-dispersible pharmaceutical formulation created could possibly alleviate some of the existing global challenges associated with the safe administration of anti-TB drugs to paediatric patients thereby promoting the achievement of desired pharmacotherapeutic outcomes (Matawo et al., 2020).

In a study, INH was used as a model drug to design and analyse a microparticulate dry suspension that can be reconstituted. The liquid and solid interphases of the drug and excipient were combined to create a homogenous mixture, which was then lyophilized and ground into an INH-loaded free-flowing powdery formulation to create the reconstitutable dry suspension. Dissolution studies were carried out on the formulation containing an equivalence of 100 mg isoniazid in pH 7.4, 6.8, and 1.2 (Adeleke et al., 2020a). Both in the dry powder form and in the hydrated form, it showed the ability to control INH release in a controlled manner and showed good stability under typical storage conditions. Considering the current worldwide shortage of such formulations, especially for primary anti-tubercular medicine, the findings from this study could contribute to improving flexible paediatric-friendly dosing for TB therapy (Adeleke et al., 2020b).

The studies mentioned above did not progress to clinical trials. Issues related to scalability, cost-effectiveness, or reproducibility of the manufacturing process can pose challenges. If a reliable and scalable method for producing the drug cannot be established, it may hinder progression to clinical trials (Desai, 2012a; Đorđević et al., 2022). Table 3 summarises commercially available formulations for the treatment of TB in paediatrics.

**TABLE 3:** Commercially available solid formulations for treatment of TB in paediatrics.

Drug	Dosage form	Manufacturer
Rifampicin 75 mg + Isoniazid 50 mg + Pyrazinamide 150 mg	Dispersible tablets	Macleods Pharmaceuticals Limited, India
Isoniazid 50 mg	Dispersible tablet	Micro Labs Ltd.
Rifampicin 75 mg + Isoniazid 50 mg	Dispersible tablets	Macleods Pharmaceuticals Limited, India
Ethambutol Hydrochloride 100 mg	Dispersible tablets	Macleods Pharmaceuticals Limited, India
Ethionamide 125 mg	Dispersible tablet	Macleods Pharmaceutical Ltd., (India)
Linezolid 150 mg	Dispersible tablet	Macleods Pharmaceutical Ltd., (India)
Linezolid 100 mg/5 mL	Granules for oral suspension	Pharmacia UK
Pyrazinamide 150 mg	Dispersible tablets	Macleods Pharmaceuticals Limited, India & Micro Labs Ltd.
Rifampicin 100 mg/5 mL	Granules for oral suspension	SW Pharma GmbH
Rifampicin 100 mg/5 mL	Granule for syrup	Avantis Pharma
Levofloxacin 100 mg	Dispersible tablets	Macleods Pharmaceuticals Limited, India

## 8 Challenges with conventional oral anti-TB dosage forms

Alternate dosage forms like suspensions or solutions, may be able to help overcome challenges with paediatric treatment, yet their application is limited due to inherent challenges. These challenges include reduced stability even when refrigerated, challenges to improve organoleptic properties, being costly to transport safely, and having short shelf lives (Ivanovska et al., 2014). Although bad tastes can be concealed by oral suspensions, there is frequently an unpleasant aftertaste, which may cause reluctance to take the medication (Alffenaar et al., 2022). On the other hand, dispersible tablets are thought to be more suitable for children, however, their usage is still restricted by the difficulty of administration while traveling or in situations of limited access to clean water. This is especially pertinent in the majority of poorly developed countries where TB is prevalent. They typically contain additional ingredients such as parabens, that are either unsafe for children to consume or have hygroscopic properties, like sorbitol, that make them prone to absorbing water from the atmosphere. The moisture absorption can lead to destabilization of the active drug, rendering it inactive, and potentially causing a decrease in therapeutic effectiveness (Singh et al., 2008). The stability issues with combining two TB drugs, such as a combination of RIF and INH, are one of the biggest obstacles in developing most of the dispersible fixed-dose combinations. Fixed-dose combination formulations of RIF and INH have the potential to generate elevated levels of INH alongside reduced levels of RIF in the stomach. This poses a significant concern as the formation of isonicotinyl hydrazone, is directly linked to the concentration of INH and the duration of exposure in acidic conditions. Substantial interaction between INH and RIF in an acidic environment may result in insufficient RIF bioavailability, potentially contributing to the development of drug-resistant TB (Bhutani et al., 2004). The use of large quantities of disintegrants is a typical method for producing dispersible tablets. Moreover, many high TB burdened countries experience elevated temperatures and high humidity levels, impacting storage conditions. The issue with several of these additives is their pronounced hygroscopic nature, which causes the tablets to gradually soften and swell spontaneously (Taneja et al., 2015a). Poor medicine taste has been shown to have a detrimental impact on children’s compliance with therapy, which in the case of TB could lead to unsuccessful therapy and encourage the rise and dissemination of MDR-TB (Baguley et al., 2012). Ion-exchange resin formation, complexation with cyclodextrins, and coating amongst others have all been used extensively as TB drug taste-masking techniques; however, these techniques frequently exhibit an unreliable ligand release rate that makes drug exposure difficult. Additionally, coating might influence the exposure and release of the active pharmaceutical ingredient (API) (Taneja et al., 2015b).

Long treatment periods and frequent, continuous dosing of multiple drugs are the therapeutic challenges that come with treating TB, causing noncompliance with current therapy. The main factor leading to the recurrence of the illness and the development of extensively drug-resistant (XDR) and MDR-TB is low patient compliance with treatment regimens (Kia et al., 2023a). While current TB medications prove their effectiveness, there is an urgent requirement to design innovative short-term regimens that integrate novel drugs to address the duration of treatment, drug resistance, and adherence to therapy. Thus, techniques to combat the therapeutic constraints of traditional treatment should be developed (Kia et al., 2023b).

Therapeutic challenges or shortcomings of using conventional dosage forms is that conventional dosage forms may fail to reach complex TB infection sites like granulomas or caseous necrotic lesions effectively. Due to their reduced blood supply and altered tissue properties, these locations can serve as reservoirs for the bacterium that causes TB and are frequently more resistant to treatment (Mhambi et al., 2021). It can be challenging to deliver adequate drug concentrations at the site of non-pulmonary infection sites using conventional dosage forms.

Among the various extrapulmonary TB manifestations, infection of the central nervous system (CNS) is notably one of the most perilous. CNS TB can manifest as spinal arachnoiditis, tuberculoma, or tubercular meningitis (Farrell et al., 2023). Anti-tuberculous drugs must enter the cerebrospinal fluid and get to the TB-infected tissue within the brain and meninges in order to be successful in the treatment of CNS TB (Norrby, 1979).

The current WHO treatment protocol for tubercular meningitis does not consider the different degrees to which anti-TB medications can penetrate the CNS (Donald, 2010). For example, several studies have reported that INH freely crosses the blood-brain barrier and plays a pivotal role as a chemotherapeutic agent in the treatment of tubercular meningitis with demonstrated potent bactericidal activity, however, RIF does not perform as effectively, with concentrations in cerebrospinal fluid (CSF) being only 10%–20% of those found in systemic circulation. Rifampicin exhibited a slow and limited entry into the CSF, maintaining concentrations slightly above the minimum inhibitory concentration (MIC) required against Mtb (approximately 0.3 mg/L) throughout the duration. The challenge of rifampicin’s penetration into the CSF was evident in the CSF/serum concentration ratios, which rose from 0.04 mg/L at 2 h to 0.11 mg/L at 6 h (Ellard et al., 1993).

## 9 Recent developments in novel TB formulations

Despite the effectiveness of the current anti-TB medications, urgent mechanisms must be established to ensure their administration. Macrophages, which are the major cells that carry Mtb, readily take up antimicrobial-loaded nanocarriers because of their size and surface chemistry. Passive targeting is a successful TB therapy method because macrophages are constantly attracted to locations where there are infections and because they can deliver drugs (Baranyai et al., 2021).

The primary advantages of nanocarrier systems compared to conventional TB drugs include improved bioavailability, protection against inactivation of encapsulated drug, enabling sustained and controlled drug release, and the potential to lower administered doses and thereby associated side effects and dosing frequency. A wide range of nanocarriers, such as nanocapsules, polymeric nanoparticles micelles, liposomes, nanogels, solid lipid nanoparticles (SLN), dendrimers and inorganic nanocarriers, have been devised to target Mtb reservoirs. Through physical encapsulation, adsorption, or chemical conjugation, therapeutic agents can be added to nanocarriers. Another significant benefit of using nanocarriers is the ability to actively or passively target host cells (Baranyai et al., 2015).

In this regard, nanotechnology offers one of the most promising avenues for the creation of more incisive and efficient pharmaceutical delivery systems for TB therapy, as well as an effective method for the creation and distribution of advanced TB vaccines. A pharmaceutical delivery system based on nanotechnology shows potential in enhancing the tolerance of chemotherapy, providing extended and regulated drug release, and achieving greater bioavailability (Nasiruddin et al., 2017b). Nanoparticles have been employed for lowering dose frequency, reducing treatment duration, increasing oral bioavailability, sustaining drug release profile, reducing administration volume and improving therapeutic effectiveness in TB treatment.

### 9.1 Lowering dose frequency

The reduction in anti-tubercular drug dosing frequency remains a therapeutic challenge. Hence, Pandey et al. (2005) designed a study to assess the potential of delivering anti-TB drugs through oral (SLN)-based on a murine TB model. RIF SLN had the highest encapsulation followed by INH then PZA using the “emulsion solvent diffusion” method. The results of the study suggested that SLNs provide a cost-effective and patient-friendly method of administering anti-TB drugs with high chemotherapeutic potential.

In order to mitigate the need for higher total doses and lengthy duration of therapy as well as to improve patient adherence, Swai et al. (2008) reformulated RIF, INH, EMB, and PZA into nanoparticulate oral formulations designed for controlled release over prolonged periods. PLGA (poly-lactide glycolide) was utilized in creating a complex nanoemulsion that underwent subsequent spray drying. This study aimed to tackle patient non-compliance in TB control programs. The drug release evaluations carried out at different pH ranges, were found to release the drugs slowly over a period of days.

A study by Ahmad et al. (2008) showcased the individual efficacy of econazole (ECZ) and (MOX) against multidrug-resistant and latent Mtb. To enhance their potential, this research assessed poly-(dl-lactide-co-glycolide) (PLG) nanoparticle-encapsulated ECZ and MOX against drug-susceptible murine TB. Prepared via the multiple emulsion and solvent evaporation technique, PLG nanoparticles were orally administered to mice. A single oral dose maintained therapeutic drug levels in plasma for up to 5 days (ECZ) or 4 days (MOX), extending to 6 days in organs (lungs, liver, and spleen), in contrast to the rapid clearance of free drugs within 12–24 h from the same organs. In mice infected with Mtb, eight oral doses of the nanoparticle formulation given weekly proved as effective as 56 doses (daily MOX) or 112 doses (twice-daily ECZ) of free drugs. Given that the two drugs target different areas, their combined action is anticipated to produce synergistic effects. Moreover, this regimen is expected to shorten the duration of TB chemotherapy and reduce dosing frequency.

### 9.2 Treatment duration

In a study by Sato et al. (2017), nanostructured lipid carriers (NLCs) were synthesised and characterised. This system was employed as a tool to assess how well three copper (II) complexes with the ligand INH might be incorporated into NLCs. All dispersions of NLCs were prepared by the melt emulsification technique. The *in vitro* efficacy of copper (II) complexes 1, 2, and 3 loaded NLCs was evaluated, along with an assessment of their *in vivo* toxicity. The technology demonstrated its promise as a tool in enhancing action against Mtb. By incorporating copper (II) complexes into NLCs intended for the oral route, TB treatment duration may be reduced, resulting in more effective therapy and improved patient compliance.

### 9.3 Increased oral bioavailability

RIF-loaded self-nanoemulsifying drug delivery system (S-SNEDDS) were prepared using an adsorption method. The ratios of Aerosil 200 (as an adsorbent) to liquid RIF-loaded-SNEDDS (as an adsorbate) were optimised to produce free-flowing, non-sticky RIF-S-SNEDDS powder. It was assessed for drug release by Hussain et al. (2019) and it was shown that they are an effective system for improving intestinal permeability and oral bioavailability. Therefore, it could be a better option for current TB therapy delivery methods.

### 9.4 Sustained drug release

For the effective treatment of MDR-TB, Kulkarni et al. (2019) created self-assembled niosomes that were dual drug-loaded with ethionamide and D-cycloserine. The niosomes were formulated using the ethanol injection method. The niosomes were characterised for osmotic shock, antibacterial investigations, *in vitro* haemodialysis, and atomic force microscopy using Box Behnken design. The haemodialysis trials demonstrated that the delivery of dual drug-loaded niosomes intravenously is safe and the formulation was suited for oral administration via mucoadhesive capsules for upcoming uses since it was stable for 6 months when refrigerated. Additionally, it had satisfactory entrapment efficiencies and maintained a sustained drug release profile for a duration of up to 3 days. When comparing pure drug and niosomes loaded with a single drug, it was observed that dual drug-loaded niosomes had the lowest MIC. This effectiveness was observed because of the rapid release of D-Cycloserine and the delayed release of the lipophilic ethambutol. As a result, the dual drug combination contained in niosomes had a synergistic effect and was a successful treatment for TB.

Gajendiran et al. (2013) synthesized a series of biodegradable, low molecular weight PLGA–PEG–PLGA tri-block copolymers in powdered form. They prepared core–shell nanoparticles (CSNPs) encapsulating the anti-TB drug INH using a sonication method followed by a water-in-oil-in-water (w/o/w) double emulsification technique. The nanoparticles exhibited drug loading efficiency ranging between 12.8% and 18.67%, with drug content varying from 6.4% to 8.9%. *In vitro* release studies demonstrated an initial burst release followed by a sustained and controlled release over an extended period. Pharmacokinetic assessments revealed that the bioavailability of INH-loaded CSNPs was 28 times higher compared to free INH. Additionally, the CSNPs exhibited sustained drug release for a prolonged duration.

### 9.4 Reducing volume of administration

In a study by Henostroza et al. (2020a), a RIF nanosuspension was developed using a miniature wet-bead milling technique to treat TB. The nanoformulation doubled the amount of rifampicin present in comparison to the commercially available product. In conclusion, the RIF nanosuspension reduced the volume of administration required in half, which could enhance patient adherence and acceptability in the long-term TB therapy in elderly and paediatric patients.

A study conducted by Ahmad et al. (2006) aimed to assess the pharmacokinetics and tissue distribution of antitubercular drugs when administered in their free form versus encapsulated in alginate nanoparticles, at varying doses in mice. Alginate nanoparticles were formulated to encapsulate INH, RIF, PZA, and EMB via controlled cation-induced gelification of alginate. The formulations were orally administered to mice at two distinct dose levels, termed D1 and D2. The study findings indicated significantly higher relative bioavailability for all drugs when encapsulated within alginate nanoparticles compared to their free forms. Encapsulated drugs sustained drug levels at or above the MIC90 in organs until Day 15 post-administration, whereas free drugs only maintained these levels up to Day 1, regardless of the dose administered. Importantly, the drug levels in various organs remained above the MIC at both dose levels for equivalent durations, demonstrating similar effectiveness. The outcomes underscore the potential of alginate nanoparticles in reducing both the dosage required and the frequency of dosing, offering a promising avenue in optimizing antitubercular therapy.

### 9.5 Taste-masking

Emerging innovative technologies in nanotechnology have shown remarkable potential for masking the bitterness of medications. The physical barrier created by nanocarriers, which encapsulate the drug and reduce or prohibit interaction of the medication with certain areas of the tongue, serves as the basis for the process of taste masking in these approaches (Nasr et al., 2022a).

Huang et al. (2018) created a method to conceal the taste of azithromycin using reverse micelles. This was achieved by subjecting a mixture of phospholipid and azithromycin to freeze-drying in medium-chain triglycerides. Azithromycin was encapsulated in medium-chain triglycerides with lecithin micelles, forming a solid barrier that effectively shields or separates the drug from taste receptors. A human taste test panel was used to assess the bitterness threshold value for the formulation. The study demonstrated that the reverse micelles successfully concealed the unpalatable taste of azithromycin.

Monteagudo et al. (2014) improved the flavour, stability, and solubility of phenobarbital by optimising its lipid-based formulation known as self-emulsifying drug delivery system. Surfactant, oil phase, co-surfactant, and water (20:4:20:56 and 20:4:35:41) were used in the chosen systems. The study showed that the selected formulations improved phenobarbital solubility, stability, and taste.

Mallappa Dandagi et al. (2014) aimed to create SLN loaded with quinine sulphate using different surfactants (Tween 80, Poloxamer 407, Poloxamer 188) through an ultrasonic solvent emulsification method. The primary goal was to conceal the bitter taste, enhancing patient adherence while providing precise dosing and a versatile system adaptable to varying body weights. The study included *in vitro* assessments of taste-masking efficiency by measuring drug release from Quinine Sulphate SLNs in simulated salivary fluid with a pH of 6.8. The findings revealed no drug release in simulated salivary fluid (pH 6.8), indicating minimal drug contact with saliva and, consequently, limited bitter taste generation. Quinine sulphate emerged as a promising candidate for solid lipid nanoparticle formulation, presenting the potential to enhance compliance among paediatric and geriatric populations by concealing the bitter taste and circumventing swallowing difficulties.

A straightforward and effective technique was devised by Panchaxari et al. to create a taste-masked oral drug delivery system capable of controlling the release of unpleasant-tasting drugs in response to pH changes within the digestive system. This system employed a pH-sensitive metal-organic coordination polymer (CPs), specifically the Fe-4,4′-bipyridine (Fe-bipy) complex, serving as a taste-masking agengt. The pH-sensitive Fe-bipy was linked to mesoporous silica nanoparticles (MSNs) housing the bitter model drug, mequindox (MEQ), within its mesopores. This linkage occurred through metal-organic coordination cross-linking, resulting in the formation of CPs-coated nanodrug MSN-NH2-MEQ@Fe-bipy. Under conditions mimicking artificial saliva (pH 6.6), the Fe-4,4′-bipyridine CPs effectively prevented the leakage of the loaded MEQ molecules. Conversely, in artificial gastric fluid (pH 1.0), the coordination bonds of the Fe-4,4′-bipyridine complex were disrupted, leading to the release of MEQ molecules from MSN-NH2-MEQ@Fe-bipy. These findings demonstrated the successful preparation of taste-masked MSN-NH2-MEQ@Fe-bipy via a perpetrating method, offering a simple and efficient approach to leveraging pH-responsive smart nanomaterials for taste-masking technology (Bao et al., 2016).

Deng et al. (2021) developed a novel nanoparticle-based method for paediatric drug delivery, with the goal of improving the oral absorption and taste of therapeutics that are both poorly water-soluble and unpalatable. They selected Lopinavir (LPV) and Ritonavir (RTV) as representative compounds due to their unpleasant taste. They created LPV and RTV Eudragit^®^ EPO nanoparticles using a nanoprecipitation technique, evaluating their essential quality traits and ability to mask unpleasant tastes. In addition, they performed *in vitro* dissolution tests that mimicked gastrointestinal pH conditions. The team used a rat model to assess the bioavailability of these nano-formulations. After optimising the formulation, they achieved over 98% encapsulation efficiency for both LPV and RTV in the nano-formulation while maintaining the amorphous nature of both drugs. Furthermore, an E-tongue study confirmed the taste-masking effect of the developed nano-formulations. These findings highlight an innovative orodispersible platform based on nanoparticles that significantly improve the oral absorption and taste of therapeutics that are unsuitable for paediatric use due to poor solubility and unpalatable nature. Furthermore, an E-tongue study confirmed the taste-masking effect of the developed nano-formulations. These findings highlight an innovative orodispersible platform based on nanoparticles that significantly improves the oral absorption and taste of therapeutics that are unsuitable for paediatric use due to poor solubility and unpalatable nature.

Nanocarrier systems offer the promising solution to address taste palatability issues, particularly for any novel drugs with a significant role in the treatment of TB. Unfortunately, anti-TB drugs have not yet been investigated for taste masking using nanotechnology. The limited exploration of nanocarrier systems for taste masking may be attributed to the intricacies of the techniques involved, the requirement for costly tools, challenges in scaling up such techniques, and the extended duration of the process, which could hinder its commercial feasibility. Nanoparticle-based delivery technologies offer a pragmatic, economical and potential alternative to TB chemotherapy (Ranjita et al., 2011). Typically, in paediatrics, it is more effective to administer drugs using oral delivery. To be effective, oral medications need to be soluble, pass through the GI mucus barrier, and sustain therapeutic concentrations in the target organs. In this regard, TB drug formulations containing NPs are being developed (Costa-Gouveia et al., 2017). The blood-brain barrier acts to inhibit drug penetration, the difficulty in treating CNS-TB lies in figuring out ways to increase anti-TB drug levels in the brain. Nanoparticles exhibit stable release, tissue penetration, and bypass of the P-glycoprotein effusion pump (Chen et al., 2020). Pandey and Khuller (2006) developed an oral nano-formulation by incorporating the anti-TB drugs INH, RIF, PZA, and EMB. The formulation was synthesized using the multiple emulsion method. A single oral dose of the formulation could sustain a therapeutic concentration both in the blood (5–8 days) and in the brain tissue (9 days). The drug doses used throughout the study were rifampicin 10 mg/kg, isoniazid 25 mg/kg, pyrazinamide 150 mg/kg and ethambutol 100 mg/kg body weight. Administering the formulation orally, in five doses, every 10th day, effectively eliminated all Mtb H37Rv from the meninges of the mice.

The information presented above indicates that nanoparticles have a significant potential for treating TB. Their key benefits, including increased drug bioavailability and decreased need for frequent dosing, could lay a solid foundation for improved disease management (Patricia Bento Da Silva et al., 2016b). Additionally, it could make directly observed treatment more cost-effective and feasible. The viability of numerous drug delivery methods, such as oral and inhalation routes, is another significant benefit of nanoparticles. Furthermore, the nanoparticles’ high stability suggests a long shelf life (Gelperina et al., 2005a).

## 10 SWOT analysis of oral nano-based drug delivery system

### 10.1 Strengths

#### 10.1.1 Critical quality attributes

Critical quality attributes refer to the physical, chemical, biological, or microbiological attributes or properties that need to fall within a suitable range, limit, or distribution in order to guarantee that the product is of the desired standard. Pharmaceutical nanocarriers offer a promising and developing method to control the therapeutic effect, release profiles, bioavailability, physical characteristics, chemical stability, and optical properties of pharmaceuticals. The acceptable ranges of CQAs are established and monitored consistently over the product life cycle. This is achieved through the application of solid scientific principles and a robust quality management system to track the performance of both the product and manufacturing process (Sarwar et al., 2019). The application of nanotechnology in the oral delivery of anti-TB drugs has several strengths and quality attributes as it has the advantage of drug carrier capacity, incorporates both hydrophilic and hydrophobic drugs into different formulations, and provides the feasibility of administration via various methods, including oral application (Gelperina et al., 2005b). The advantages of using nanoparticles also include greater mucoadhesion, increased retention in the GI system, and protection of TB drugs, from degradative enzymes. In the digestive tract, polymeric nanoparticles may function as bioadhesives. The benefits of using bio/mucoadhesive drug delivery systems are: 1) extended period at the targeted site, thereby enhancing drug bioavailability; 2) it is possible to deliver the drug at a particular site or tissue; and 3) extended residence time, which, when coupled with long-acting drugs, can potentially result to reduced need for frequent administration (Woodley, 2001). The incorporation of ligand, often referred to as a bioadhesive ligand, has further enhanced polylactide-co-glycolide (PLGA) nanomedicine. A mucosal ligand called lectin has additionally shown improved nanoparticle adhesion to the mucosal surface, increasing anti-TB drug (RIF, INH, PZA) absorption and bioavailability (Gabor et al., 2004). Utilizing nanoparticles to enhance mucoadhesion offers the advantage of boosting the oral administration of anti-TB agents that are weakly adsorbed. This is achieved by lengthening the duration and extent of interaction with the intestinal mucus layer of the intestine (Laura et al., 2012). For example, micelles which are colloidal carriers (5–100 nm) are created to increase the drugs’ water solubility and ease oral delivery. Furthermore, micelles can enhance the active penetration of drugs in addition to passive penetration (Dabholkar et al., 2006). On the other hand, liposomes are able to encapsulate both hydrophobic and hydrophilic drugs inside their respective hydrophobic compartments and inner hydrophilic cores (Vladimir, 2005).

Oral drug delivery systems such as nanoparticles are imperative to enhance drugs for administration orally as well as to promote sustained targeted delivery and co-delivery of drugs (Homayun et al., 2019). Nanoparticles could, in fact, address many of the needs associated with paediatric medications, this is due to their ability to: 1) modify drug properties 2) taste masking which makes the taste of drugs more palatable to young patients 3) simplify drug administration 4) to achieve an extended drug release, reducing the number of doses required and improving both patient and parental compliance; 5) when targeting drugs; 6) to make it simple to adjust dosages as children grow; 7) and to make it possible to combine more medications in one formulation (González et al., 2021). Because of the stability and controlled delivery of the drugs from nanoparticles, oral administration is made possible (Pandey and Khuller, 2004).

#### 10.1.2 Critical process parameters

Final production process parameters include the identification of Critical Process Parameters (CPPs), and the risk evaluation of the drug formulation which contributes to the final product (Yenduri et al., 2022a). The ability to manufacture nanoparticles using a variety of techniques allows for greater flexibility in the formulation process, resulting in nanocarriers with the desired pharmaceutical properties. In the study of polymer nanoparticles, a variety of encapsulation techniques have been investigated, including mini-emulsion, single emulsion, double emulsion, polymerisation, nanoprecipitation supercritical fluids, spray drying, organic phase separation, and solvent diffusion. Among these techniques, emulsion and solvent evaporation techniques are straightforward, easy to implement, fast, and adaptable for large scale production (Panigrahi et al., 2021).

#### 10.1.3 Critical material attributes

The term “critical material attribute” (CMA) refers to a material whose variability affects the CQAs and should be tracked or managed to ensure that the desired quality target product profiles are reached (Yenduri et al., 2022b). Polymeric nanoparticles are simple to synthesize, but their stability and choice of stabiliser(s) is the most difficult and critical step. Polymers have been used to shield acid labile drugs from undergoing degradation by the effect of acidic environment or gastrointestinal enzymes, to reduce irritation of gastric mucosa caused by some drugs, and to achieve target-specific drug delivery by delivering drugs selectively to the site of absorption (Ahmed et al., 2015). The therapeutic efficacy of polymeric nanoparticles is typically influenced by their physicochemical characteristics which include factors such as size, shape, zeta potential, loading capacity, and surface functionality with appropriate surfactants (Voigt et al., 2014). Surfactants, amphiphilic molecules with an ionic or non-ionic hydrophilic head group and a lipophilic tail, are essential excipients in the manufacturing of nanoparticles. Hydrophobic nanocarriers have been stabilized in aqueous media by using surfactants’ amphiphilic properties (SamyJoohoon et al., 2020). Non-ionic surfactants are widely used in the production of polymeric nanoparticles due to their excellent biocompatibility and minimal interaction with biological barriers (Cortés et al., 2021).

Nanocarriers can serve multiple functions at once, such as treating the target disease and imaging it using theranostic nanoparticles. In order to minimize adverse effects, therapists can facilitate site-specific drug delivery, monitor therapy noninvasively, and diagnose Mtb infections at earlier stages (Kia et al., 2023c). Theranostic agents are nanoscale therapeutic systems with multiple functions, which include diagnosis, targeted drug therapy, and observing how well patients respond to the therapy. It allows for tracking the drug’s release, distribution within the body, and accumulation at the target area, as well as dose adjustments for specific patients and, finally, tracking the progression of an infection (Wyszogrodzka et al., 2018).

### 10.2 Weaknesses

Despite many features that make nanotechnology a viable and attractive application for oral drug delivery for TB drugs, there are key limitations that could impede their potential for effective development into marketable pharmaceutical products. Firstly, formulation of nanoparticles specifically for children have always been avoided due to the intricacies of clinical trials in children, which arise from scientific, medical, ethical, procedural, and operational issues (Bankole, 2006). As such, the number of research initiatives dedicated to the development of nanotechnology-driven anti-TB medicines for children in particular, is woefully low (Sosnik and Angel, 2014). Only a small number of research teams globally, mostly in underdeveloped nations, have concentrated on creating paediatric anti-TB treatments using nanotechnology (Sosnik et al., 2010b).

For instance, Khuller and Swai’s team conducted research into the encapsulation of various anti-tubercular medication using polymeric and lipid nanoparticles. They assessed the oral effectiveness of these formulations in TB infected mouse models (Sharma and Khuller, 2004). However, there are hardly ever designed to cater for the paediatric population. Secondly, another challenge is nanomedicines’ unique complexity and multicomponent structure add a lot of new variables, which may significantly increase how difficult it is to regulate processes and predict behaviour in biological systems. When generic nanomedicines are submitted for health authority authorization with claims of equivalence to the innovator drug, additional regulatory and development factors emerge (Desai, 2012b).

The toxicity of nanoparticles is dependent on a number of variables, including particle size, shape, surface chemistry, and composition. It would be difficult to translate these nanoformulations into clinical treatments because some biological reactions, like the enhanced-permeability and retention effect, might not correlate in human subjects (Paliwal et al., 2014) Limited training and education about nanoparticles, their mechanisms, cost-effectiveness and accessibility, and appropriate application in paediatric medicine might lead to reluctance among medical teams to integrate these innovations into clinical practice (Paliwal and Palakurthi, 2014; Hutchison, 2016a).

### 10.3 Opportunities

Evidently, Liposomes, nanoparticles, nanosuspensions, nano-structured lipid carriers, and nanoemulsions are all forms of nanocarrier systems that are used in nanotechnology for anti-TB oral drug delivery (Henostroza et al., 2020b). Numerous nanocarrier systems have been developed, each of which offers the benefit of successful taste masking while maintaining the drug solubility and release properties. Nanocarriers such as liposomes (Tang et al., 2017), solid lipid nanoparticles (Zhang et al., 2020), polymeric nanoparticles (Naik et al., 2021), nanostructured lipid carriers (Zardini et al., 2018), polymeric micelles (Pan et al., 2020), submicron lipid emulsion (Hasan et al., 2015), nanosponges (Omar et al., 2020), and pH-responsive polymers have been used in the taste masking of various drugs.

Pharmaceutical product manufacturers, however, have not yet given nanocarrier systems for taste masking applications in anti-TB drugs enough attention. Further developments of nanocarrier systems, as well as additional research, are required to use these techniques on various bitter taste molecules. In a paper, Nasr et al. (2022b) reviewed a variety of taste-masking strategies utilising nanocarrier systems, highlighting the make-up of these systems and their use in developing oral therapeutic delivery systems for molecules with a bitter taste. Despite producing effective results, there is little research being done on the use of nanocarrier systems for taste masking of anti-TB drugs, according to the data gathered for the review. Researchers and the pharmaceutical industry will be able to create novel drug delivery systems with improved taste-masking capabilities with the aid of a better understanding of the fabrication of nanotechnology-based drug delivery systems (Vishvakarma et al., 2022). The positive outcomes of the studies conducted with nanoparticles *in vitro* and *in vivo* provide an encouraging foundation for further investigation and potential clinical applications in TB treatment.

Due to first pass metabolism and drug interactions caused by the presence of multiple transporters and metabolising enzymes in intestinal epithelium, oral medications cause decreased bioavailability that can be prevented by long-acting nano-formulations (Dhanashree and Jindal, 2020). Long-acting formulations have been very effective in a variety of medical fields, including psychiatry, birth control, and most recently, HIV/AIDS treatment. Although difficult, using this technology for TB therapy could have a significant impact (Swindells et al., 2018).

The crucial properties of a molecule that must be taken into account for effective therapeutic effects in the context of oral drug delivery are solubility and ionization, lipid affinity and permeability, stability in biological fluids, and gastrointestinal metabolism. Nanocarriers like polymeric nanoparticles, dendrimers, and polymeric micelles offer the capability to encapsulate drug molecules for solubilisation, controlled release, targeting specific sites, or protection from the gastrointestinal environment (Sharma and Garg, 2010). Because of advantages such as improved drug bioavailability, reduced dosing frequency, and increased stability, nanoparticles have significant potential for TB treatment (Hutchison, 2016a).

Interest in pulmonary drug delivery is another alternative that is increasing because of its benefits, which includes non-invasive administration, direct penetration into lung tissues, bypassing hepatic first-pass metabolism, lower enzymatic activity for drug metabolism, quick onset of action, and high drug bioavailability in the bloodstream (Prakash et al., 2018). Utilizing inhaled therapy with both new and existing drugs could provide an additional method to manage drug resistance while more comprehensive strategies are developed (Hickey et al., 2016).

Aerosol technology advancements now allow for precise dosage and deposition, highlighting this method for targeted treatment of airway infections. While many inhalation devices are designed for adults, adapting them for paediatric use necessitates changes to accommodate the anatomical and physiological characteristics of various paediatric subgroups, as well as regulatory approval. For example, combining a spacer system and a face mask could allow infants to use pressurised metered dose inhalers from birth (European Medicines Agency, 2006). On the other hand, dry powders would be suitable for use in children beyond the age of 1 year. Pandey and Khuller (2005) investigated this technique for delivering polymeric and lipid nanoparticles to alveolar macrophages, with the goal of targeting *mycobacterium*’s intracellular reservoir.

### 10.4 Threats

Nanomedicines continues to explore novel drugs and expand its application to address different disease indications while gaining more traction in the clinical setting. In addition to keeping an eye on developments from a secure academic vantage point, the nanomedicine research community should make every effort to picture their implementation in clinical practice.

With that being said, if the current anti-TB formulations are straightforward, low-cost and the API has evolved into a specialized and advanced nanomedicine product, the primary question that arises: will doctors and patients be willing to switch from the conventional anti-TB treatment to a new approach that significantly increases the overall treatment cost (Josbert and Lammers, 2020).

Secondly, the initial development of nanomedicines frequently takes place in the lab, and there is a big difference between academic research and commercial production. In academic laboratories, products are typically produced in microgram or milligram quantities, whereas pre-clinical testing, clinical trials, and ultimately clinical use require grams or kilogram quantities (Longo et al., 2020). Any laboratory process can be challenging to scale up, but producing nanoparticles is particularly difficult because even small changes in the manufacturing process can have a significant impact on the properties of the finished product, which ultimately determine the therapeutic outcome (Desai, 2012c).

**FIGURE 2:**
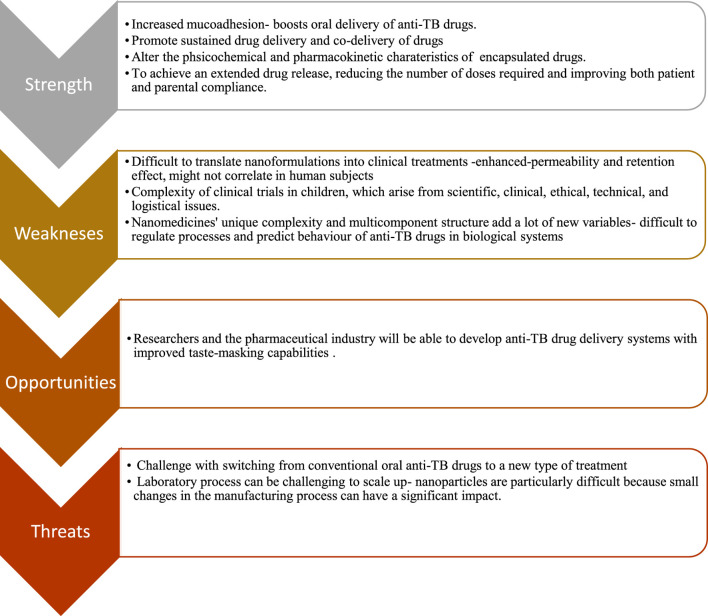
SWOT Analysis summary of oral nano-based drug delivery system.

Another potential threat to the success of nanomanufacturing is the training of the personnel on the details, obstacles, and challenges associated with product manufacturing (Ragelle et al., 2017). A summary of the SWOT analysis of oral nano-based drug delivery systems is provided in Figure 2.

## 11 Future perspectives

Choosing the most suitable formulation design and excipients should be based on a comprehensive consideration of factors such as patient safety, manufacturability and end-user preferences including aspects like taste and ease of administration. Over the last two decades, a significant amount of research, development and patenting efforts have been dedicated to age-appropriate pharmaceutical products with some successfully gaining marketing authorization. Nonetheless, strategies for creating paediatric-friendly oral drug delivery systems have remained relatively underdeveloped. With the maturity of novel TB therapy, more and more nanoparticle-based drugs are also being studied for development to improve outcomes for adults with TB. Nonetheless, effective strategies for the treatment of TB in paediatrics remain elusive and only a few drugs are now approved for paediatric patients.

A number of nanoparticulate systems, including sublingual and buccal drug delivery, have been studied, with polymer- and lipid-based formulations being the most common. Due to the ease of administration and high level of compliance associated with oral administration, nanotechnology has been extensively researched in oral drug delivery. Nanofibers that have been loaded with drugs have recently attracted interest as potential systems for drug delivery due to their distinctive structure.

After a thorough review of various studies, it becomes evident that researchers must prioritize addressing the critical shortage of nanocarrier-based oral drug delivery used in TB. The current lack of clinical trial protocols is one of the biggest obstacles hindering the advancements of paediatric nanomedicine. As nanotechnology gains wider acceptance and more nanotechnology-related products are developed, the use of nanomedicine applications for treating paediatric TB is anticipated in the near future. Niosomes and liposomes have undergone extensive research for the purpose of treating paediatric diseases, but only a limited number are employed in therapeutic applications and treatment of TB.

## 12 Conclusion

In this review, we can outline that the main challenge with the treatment of paediatrics falls in the lack of research and literature work done on paediatric-friendly formulations. Formulations that meet all the criteria for an ideal anti-TB paediatric medication are limited due to a lack of extensive studies in paediatrics particularly. The present study aimed to explore gaps in TB medicine formulation in the conventional field and in nanotechnology.

Continuous technological advancements require relevant patient outcome studies and clinical input on the effectiveness, safety, patient satisfaction, preferences, and compliance regarding new compositions; currently, these studies and feedback are deficient. Experience in paediatric TB has shown that the current separate work streams for developing and implementing paediatric formulations are insufficient to produce the best formulations for children, and a more organised and effective collaboration is needed.

The continued prioritisation of unaddressed formulation needs, specifically pertaining to drug delivery in paediatrics and treatment deficiencies in paediatric TB, is a critical issue that needs to be tackled to encourage continued advancements of improved medications for young patients. Emerging nanoparticle-based delivery technologies offer a feasible, and promising choice for TB chemotherapy. Better drug bioavailability, dose frequency reduction, viability of several drug administration methods, and long-term stability may all be the foundation for more effective disease management in TB.

The future of oral nanoparticles in treating TB in paediatrics holds substantial promise and potential advancements. However, challenges like regulatory approvals, scalability of production, and long-term safety need to be taken into consideration for the widespread adoption of nano-formulations in TB treatment in the paediatric population. Collaborative efforts among researchers, healthcare providers, regulatory agencies, and pharmaceutical industries will be crucial in realizing the potential of nano-formulations for paediatric TB treatment.
